# The interplay between spin densities and magnetic superexchange interactions: case studies of mono- and trinuclear bis(oxamato)-type complexes

**DOI:** 10.3762/bjnano.8.224

**Published:** 2017-10-27

**Authors:** Azar Aliabadi, Bernd Büchner, Vladislav Kataev, Tobias Rüffer

**Affiliations:** 1 Leibnitz Institute for Solid State and Materials Research, IFW Dresden, Helmholtzstrasse 20, D-01069 Dresden, Germany; 2 Institut für Festkörperphysik, TU Dresden, Zellescher Weg 16, D-01062 Dresden, Germany; 3 Department of Inorganic Chemistry, Faculty of Natural Sciences, TU Chemnitz, Strasse der Nationen 62, D-09111 Chemnitz, Germany

**Keywords:** bis(oxamato), bis(oxamidato), copper(II), electron nuclear double resonance, electron paramagnetic resonance, magnetic superexchange interactions, pulsed electron–electron double resonance, spin density distribution

## Abstract

For future molecular spintronic applications the possibility to modify and tailor the magnetic properties of transition-metal complexes is very promising. One of such possibilities is given by the countless derivatization offered by carbon chemistry. They allow for altering chemical structures and, in doing so, to tune magnetic properties of molecular spin-carrying compounds. With emphasis on the interplay of the spin density distribution of mononuclear and magnetic superexchange couplings of trinuclear bis(oxamato)-type complexes we review on efforts on such magneto-structural correlations.

## Introduction

The flexibility of carbon chemistry together with the structural variety of coordination chemistry offers unique possibilities to design new coordination complexes. This includes the potential of metalloligands for a metallosupramolecular perspective [1–7]. The genesis of this field with respect to modern developments of molecular magnetism has been comprehensively reviewed recently [8]. The multidisciplinarity of the field is vividly demonstrated by reference to, for example, electro- and/or photoswitchable complexes as active magnetic components for future applications in information processing and data storage [8]. To that development, namely the design of novel metalloligands for the synthesis of multinuclear, multidimensional and multifunctional magnetic materials we did already contribute. For example, we reported on the first chiral bis(oxamato)-type metalloligand [9], later on used by Ferrando-Soria et al. for the design of the first chiral single-chain magnets (SCMs) [10]. Additionally, we reported how to introduce the redox-active anthrachinone functionality into bis(oxamato)-type metalloligands [11], later on adapted for the design of higher nuclear complexes that could potentially act as molecular magnetic capacitors [12], or we reported to which extent a ferrocene group in multinuclear bis(oxamato)-type complexes is suited to vary magnetic properties with respect to its oxidation state [13].

**Scheme 1:**
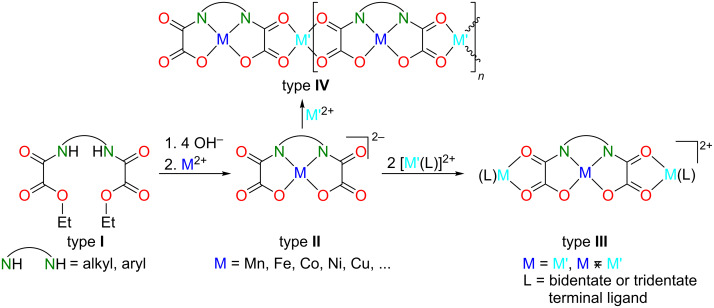
Chemical structures of type **I**–**IV** species and principal synthetic strategy to obtain type **II**–**IV** complexes. Additionally coordinated apical donors of the metal ions are not displayed.

Among many different types of metalloligands [8], the already mentioned archetypal bis(oxamato)-type complexes (type **II**, Scheme 1) are just one, but a very versatile, representative. From their precursors, usually the diethyl ester of *N,N*’-bridged organodiyl(oxamic acid) denoted as type-**I** molecules in Scheme 1, type-**II** complexes are comparatively easily accessible. The first example for such a type-**I** molecule was reported by Gaade in 1936 [14], while the capability of type-**II** complexes to act as metalloligands has been reported for the first time by Monoyama et al. [15] in 1976. Intriguingly, this first report of type-**II** complexes stated that “[…]The oxamide moiety bridging two metal ions […] serve as a pathway through which electron spin interactions takes place and their copper complexes […] are magnetically subnormal[…]” [15]. The beauty of this “bottom-up” approach, i.e., the addition of transition-metal complex fragments and transition-metal salts to type-**II** metalloligands to obtain discrete trinuclear and 1D polynuclear complexes (type **III** and **IV**, Scheme 1), respectively, or the synthesis of multifunctional 2D and 3D networks with potential applications in information storage, nanotechnology, molecular electronics and spintronics was impressively brought into bloom by O. Kahn himself and his school later on [5,8,16–18]. Type-**III** complexes are of interest because, for example, the determination of their magnetic properties, in particular their magnetic superexchange couplings, is an estimate of the magnetic properties of higher nuclear complexes [5,8,16–18]. As a strict orthogonality of magnetic orbitals cannot be achieved for higher nuclear complexes derived out of type-**II** complexes, they always possess an antiferromagnetic superexchange coupling. However, an alternative strategy is offered when heterometallic type-**IV** complexes combine paramagnetic metal ions with a large and a small spin quantum number. In such a case the magnetic ground state will be ferrimagnetic. In doing so, Kahn and his school [19–20] gave access to the first SCMs, see above, a class of magnetic material that exhibits a slow relaxation of the magnetization below the blocking temperature.

Oxamato-based SCMs, especially when equipped with further redox-switchable functionalities, are regarded as novel materials for the design of molecular spintronic devices [8,17–18]. Into such devices [21–22] diamagnetic molecules [23] and even individual single-molecule magnets (SMMs) [24] were already successfully integrated, and spin-organic field-effect transistors [25] or spin-organic light-emitting diodes [26] were developed. Although it remains puzzling to understand the spin-polarized transport phenomena of spintronic devices in detail, we focused on the synthesis of type-**III** complexes as models of SMMs with the aim to deposit them as thin films on surfaces. Already in 2006, we reported on the deposition of thin films of a type-**III** complex by spin coating [27], although the surface roughness of the thin films prevented any reliable characterization by magneto-optical Kerr effect (MOKE) spectroscopic studies. Spectroscopic MOKE measurements of such thin films would be, from a rather fundamental point of view but also with respect to potential applications of SMMs and SCMs, very interesting as magneto-optical effects are used in various optoelectronic devices [28]. It took us roughly a decade to understand how to tailor type-**III** complexes to engineer from them smooth thin films them suitable for spectroscopic MOKE studies [29], but this odyssey is not content of this review.

Along with these efforts we became interested in possibilities to strengthen and to tailor the *J* couplings of type-**III** complexes as well as to identify their magnetic superexchange pathway itself. Such an understanding is one prerequisite for the rational design of new molecular multifunctional magnetic materials for material science applications. Moreover, it would be fascinating if electron spin resonance (ESR) spectroscopic studies could give access to the spin density distribution of type-**II** complexes as an experimentally achievable measure of the magnetic superexchange couplings of type-**III** complexes and of related higher nuclear magnetic materials. There are already reports of spin and electron density distribution studies by polarized neutron and high-resolution X-ray diffraction measurements, respectively, for a ferrimagnetic type-**IV** coordination polymer [30–31]. Both studies revealed formidable direct experimental evidence that “[…]the oxamato bridge […] exhibits spin delocalization, responsible for exchange coupling along the chains[…]” [31], as anticipated by Monoyama et al. [15]. The electron density study pointed out that there is a larger spin delocalization along the N
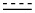
C
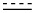
O compared to the O
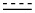
C
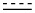
O part of an individual oxamato-bridging unit [31]. This was understood as an indication that the magnetic superexchange pathway preferably went along the N
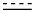
C
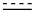
O part. Consequently, the authors did conclude according to O. Kahn [32], that “[…]Replacing all O atoms of an oxalate group by N atoms (or even better by S atoms) should therefore induce higher exchange J couplings[…]” and that comparative and systematic studies should be carried out including oxamato-, oxamidato- and thio-oxalato-bridged complexes to better understand the magnetic superexchange interactions mechanisms and to classify them topologically [31]. In the following we aim to review on efforts to determine the spin density distribution of mononuclear type-**II** and related complexes by ESR spectroscopy as a measure of the magnetic superexchange interactions of their related trinuclear type-**III** complexes.

## Review

### The concept

The spin density of the N
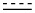
C
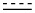
O part exceeds significantly the one of the O
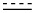
C
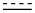
O part of type-**IV** complexes, as demonstrated experimentally [30–31]. Hypothetically, this situation can be assumed for type-**III** complexes as well. Moreover, one could hypothetically assume that the spin densities of the N atoms and the paramagnetic metal ions of type-**II** complexes as precursors of type-**III** complexes are a direct measure of the magnitude of *J* couplings. Hence, the larger the spin densities at the N atoms and the smaller at the metal ions of type-**II** type complexes, the larger the magnitude of *J* couplings of corresponding type-**III** complexes and vice versa. Spin densities or the spin density distribution, respectively, of paramagnetic transition-metal complexes can be determined by making use of ESR spectroscopy. This method in its continuous wave [33] and, in particular, in the microwave-pulse versions [34–41] has a long history of applications in this research field. By now ESR has become an established method along with neutron diffraction (ND) and nuclear magnetic resonance (NMR) spectroscopies since it does not require large amounts of a sample as is the case for ND, and often offers a better sensitivity than NMR spectroscopy. One possibility to investigate the electron spin density by ESR is, as nicely described in [33], the fabrication of single crystals composed of the paramagnetic complex of interest co-crystallized in the host lattice of a corresponding and structural analogous diamagnetic complex, which usually should be even isomorphic. The single crystals itself should be large enough in order to be able to manipulate them reliably. In an initial study we co-crystallized a Cu(II)-containing type-**II** complex in the host lattice of the corresponding Ni(II) complex [42]. In doing so, we managed to obtain diamagnetically diluted single crystals. X-band ESR studies gave access to all components of the g-factor tensor, the tensors of on-site ^Cu^*A* and transferred ^N^*A* hyperfine interactions [42]. The orientation of the single crystals within the ESR spectrometer is of crucial importance, as for certain orientations the spectra become complicated due to small ^63,65^Cu hyperfine couplings overlapped by ^14^N quintets. Especially in case of arbitrary *B*_0_ orientation the N atoms are not magnetically equivalent and this may result in less resolved triplets of triplets for the ^14^N hyperfine patterns [42]. The obtained experimentally derived spin density distribution of the Cu(II)-containing type-**II** complex compares excellently with values derived out of quantum chemical calculations [42]. In a subsequently performed study we investigated seven different Cu(II)-containing type-**II** complexes and determined their spin density distribution by X-, Q-, and W-band ESR studies [9]. With the access to Q- and W-band ESR spectrometers the fabrication of diamagnetically diluted single crystals is not a prerequisite anymore to extract the required information. Instead, diamagnetically diluted powders could be shown to be sufficient for this purpose [9], since at Q- and especially at W-band frequencies the powder pattern of the ESR spectrum arising due to the g-factor anisotropy is much better resolved. We demonstrated that the higher the tetrahedral distortion of the CuN_2_O_4_ coordination units of type-**II** complexes is, the larger is the spin density at the Cu(II) ions and the smaller it is at the N atoms. Consistently, our study strongly suggests that the magnitude of the *J* coupling of a certain type-**III** complex is larger, the smaller the spin density at the Cu(II) ion and the larger at the N atoms of the corresponding type-**II** complexes are [9].

**Figure 1:**
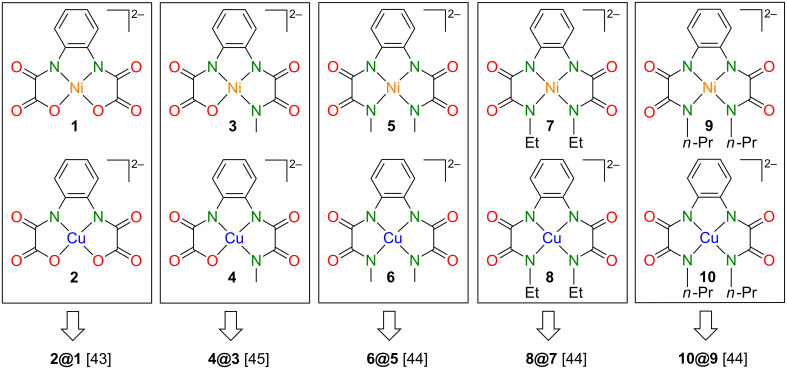
Chemical structures of reviewed pairs of diamagnetic Ni(II) and the corresponding Cu(II) complexes.

Now, we turn towards the pairs of structurally related mononuclear complexes displayed in Figure 1 and to compare the obtained results with the one reported for the combination of **2@1**. As Figure 1 illustrates, we successively replaced O atoms with N–R units. If otherwise identical to type-**III** complexes the trinuclear complexes derived out of **4**, **6**, **8** and **10** should have larger *J* couplings according to [31]. Furthermore, in case that the interplay between the spin density distribution of mononuclear and the *J* couplings of trinuclear complexes is of general validity, the spin densities at the Cu(II) ions should decrease and those of the N_aryl_ atoms should increase when going from of **2** over **4** to **6**, **8** and **10**.

### Fabrication of diamagnetically diluted single crystals

**Figure 2:**
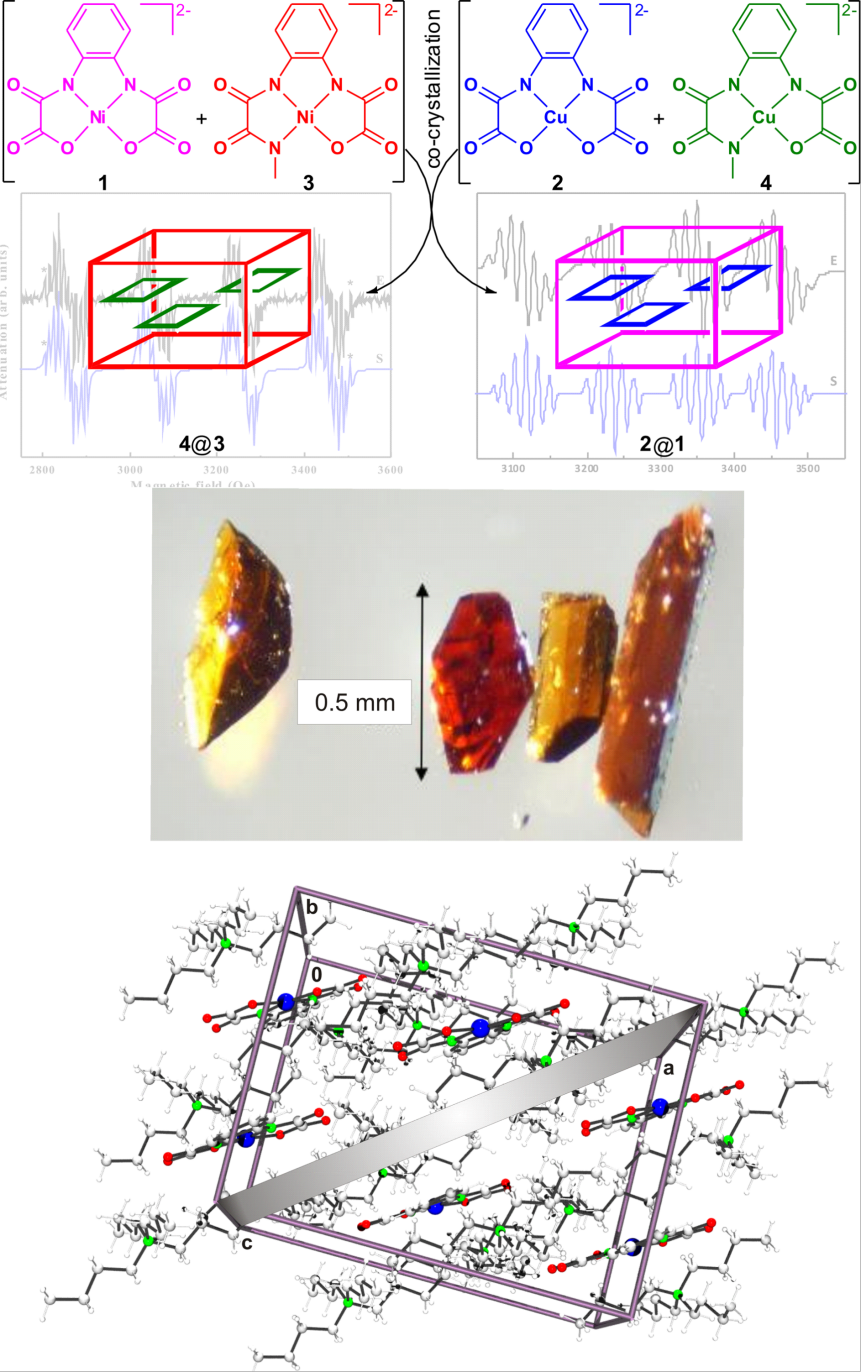
Top: Schematic representation of the successful co-crystallization of a mixture of two different Cu(II) (**2**, **4**) and two different Ni(II) containing complexes (**1**, **3**). Middle: Single crystals of diamagnetically diluted **4**@**3** (left) and **2**@**1** (right). Reproduced with permission from [44], copyright 2013 The Royal Society of Chemistry. Bottom: Cut-off of the crystal structure of **1**, displaying the orientation of the complex fragments with respect to the (101) plane. Color code: Cu (blue), O (red), N (green), C (light grey), H (white), cell truncate (purple) and (101) plane (grey).

Single crystals of **2@1**, **4@3**, **6@5**, **8@7**, and **10@9** (Figure 1) required for the ESR studies could be obtained in the same way as those of the individual complexes. There are no additional arrangements to be made. It seems likely, that the isostructural Ni(II) and Cu(II) complexes should be isomorphic as well in order to obtain diamagnetically diluted single crystals. Based on our experiments performed so far we cannot verify this further but aim to indicate that **9** and **10** crystallized in different monoclinic space groups, although their measures were nearly identical [43]. Furthermore we reported that single crystals of **6@5** were too small to be suitable for ESR studies, although both complexes could be crystallized individually in form of very large single crystals [43]. Additionally we noticed with surprise [44], that the crystallization of a mixture of the complexes **1**–**4** resulted in the formation of **2@1** together with **4@3** as a remarkable example for the supramolecular recognition of isostructural complexes (Figure 2). For a better comprehension, Table 1 reports on selected crystallographic data of **1**–**10**.

**Table 1:** Selected crystallographic data of **1**–**10**.

	unit cell parameters						volume (Å^3^)	crystal system	space group
	*a* (Å)	*b* (Å)	*c* (Å)	α (°)	β (°)	γ (°)			

**1** [30]	18.5088(4)	17.1731(4)	14.2230(4)	90.0	91.997(3)	90.0	4518.1(2)	monoclinic	*C*_2_/*c*
**2** [30]	18.5716(9)	17.2023(7)	14.1556(5)	90.0	91.897(4)	90.0	4519.9(3)	monoclinic	*C*_2_/*c*
**3** [30]^a^	11.3391(3)	13.9271(4)	15.9078(5)	97.566(3)	95.666(3)	110.349(3	2306.5(1)	triclinic	*P*−1
**4** [30]^a^	34.213(1)	13.3036(4)	19.7816(5)	90.0	90.0	90.0	9003.8(4)	orthorhombic	*Pna*2_1_
**5** [29]	10.7141(4)	14.4059(5)	15.4535(6)	99.540(3)	90.910(3)	102.522(3)	2292.9(2)	triclinic	*P*−1
**6** [29]	10.583(13)	14.534(3)	15.609(15)	98.444(11)	91.564(9)	102.01(1)	2318.8(5)	triclinic	*P*−1
**7** [29]	24.3834(7)	13.4528(3)	15.9421(4)	90.0	110.328(3)	90.0	4903.7(2)	monoclinic	*C*_2_/*c*
**8** [29]	24.396(5)	13.432(3)	15.919(3)	90.0	109.80(3)	90.0	4908(2)	monoclinic	*C*_2_/*c*
**9** [29]	13.5126(5)	14.7246(4)	25.4056(7)	90.0	95.567(3)	90.0	5031.0(3)	monoclinic	*P*2_1_
**10** [29]	13.4875(6)	14.6748(7)	25.6140(12)	90.0	95.346(4)	90.0	5047.6(4)	monoclinic	*P*2_1_/*c*

^a^**3** was crystallographically characterized as [*n*-Bu_4_N]_2_[Ni(opooMe)]·1.25H_2_O, while **4** was characterized as [*n*-Bu_4_N]_2_[Cu(opooMe)] [44], although **3** and **4** were crystallized under identical conditions. As a consequence the isostructural compounds **3** and **4** are not isomorphic. Single crystals of **4@3** were checked to correspond to the measures reported for **3**.

### Experimental determination of spin densities

The relevant interactions that determine the parameters of the Cu(II) ESR spectrum can be described by the following standard Hamiltonian [9,43,45]:

[1]
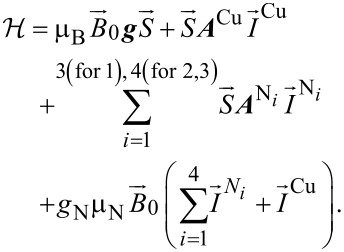


Here, the first term represents the Zeeman interaction of an electron spin *S* with an external magnetic field *B*_0_, while ***g*** and μ_B_ stand for the g-tensor and the Bohr magneton, respectively. The hyperfine (HF) interaction between the electron spin *S* of Cu(II) and the ^63^Cu, ^65^Cu and ^14^N nuclear spins *I*^Cu^ and *I*^N^ is described by the second and the third term, respectively. Here, ***A***^Cu^ and ***A***^N^ are the on-site Cu and transferred N HF coupling tensors, respectively. The last term describes the nuclear Zeeman interaction of the ^63^Cu, ^65^Cu and ^14^N nuclear spins *I*^Cu^ and *I*^N^ with the external magnetic field *B*_0_, which is not included for the modelling of continuous wave (CW) ESR spectra. Finally, *g*_N_ and μ_N_ denote the nuclear *g*-factor and the nuclear magneton, respectively. CW ESR measurements were performed at room temperature with a Bruker EMX spectrometer operating at the X-band (10 GHz) frequency. ESR spectra were processed using the Win-ESR^®^ software package [46]. Isotropic ESR parameters of the studied compounds were obtained from measurements of the samples dissolved in MeCN at a concentration of 1 mM. The angular dependence of the ESR spectra of the single crystals under study (Figure 1) was measured by rotation of the magnetic field *B*_0_ in the plane perpendicular to the molecular plane to obtain anisotropic ESR parameters. A standard manually controlled goniometer from Bruker was used for this purpose with the sample attached to a quartz-rod sample holder.

**Figure 3:**
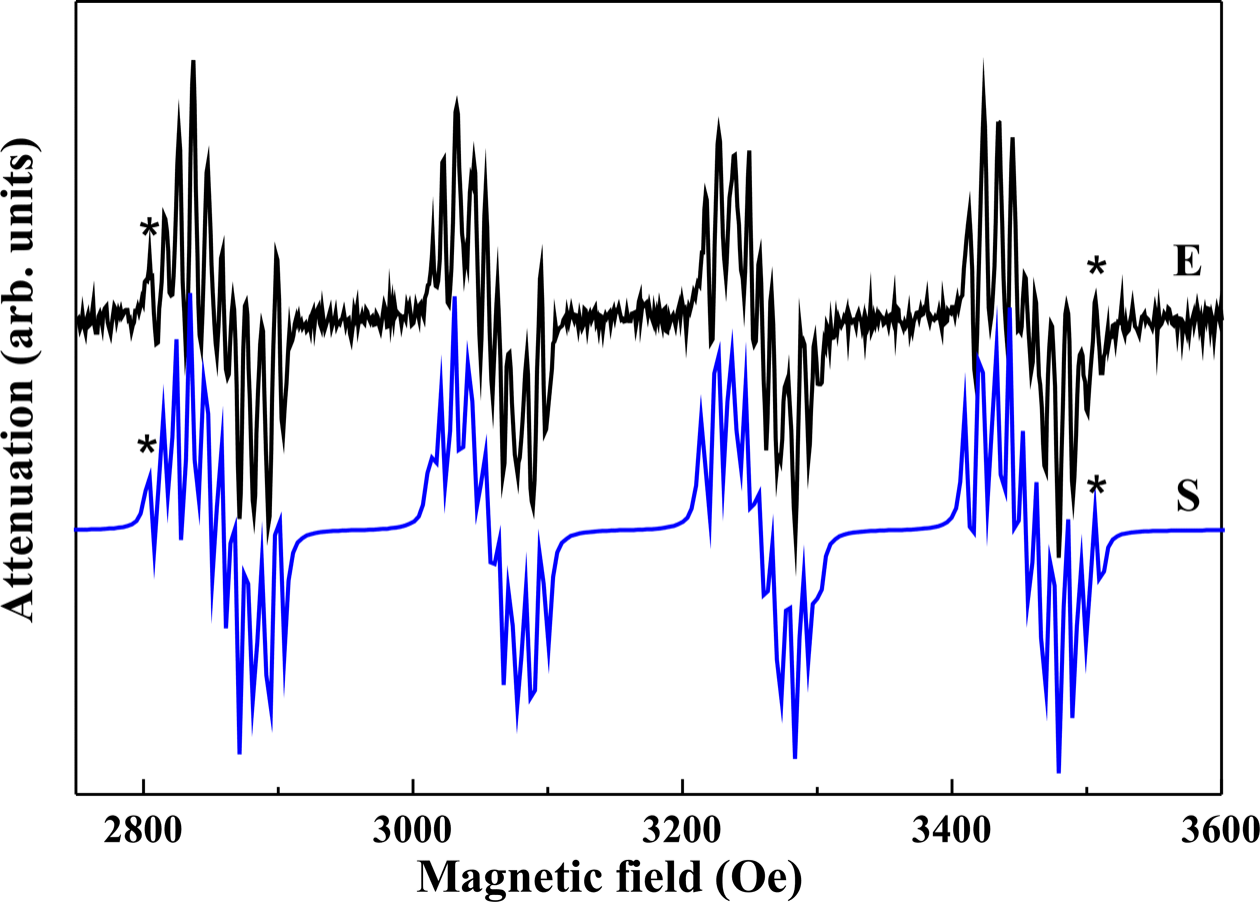
Experimental (E) and simulated (S) X-band ESR spectra of **4**@**3** at 90° orientation (*B*_0_ 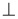 molecular plane). Reproduced with permission from [45], copyright 2012 The Royal Society of Chemistry.

As an example, experimental and simulated X-band ESR spectra of **4**@**3** at 90° orientation (*B*_0_ 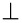 molecular plane) reported in [45] are shown in Figure 3. This preferred orientation can be judged conveniently when performing a crystallographic face-indexation of the diamagnetically diluted single crystals (Figure 2). The spectra consist of a quartet of groups of lines owing to the on-site HF coupling of an electron spin of *S*(Cu(II)) = 1/2 to its own nuclear spin *I*(^63,65^Cu) = 3/2. Each group further represents a subset of lines arising due to a transferred HF**-**coupling with the ^14^N nuclear spins *I*(^14^N) = 1 of the N donor atoms. Due to a large line width, the lines from the transferred HF coupling of an individual N-donor atom are not well resolved. For *B*_0_ parallel to the molecular plane the line groups overlap because of the small ^63^Cu HF coupling constant, 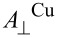, in this direction. Therefore, the extraction of the coupling parameters gets very difficult. Indeed, an estimate of 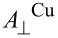 can be obtained by using the relation 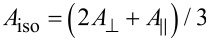, where the isotropic HF constant *A*_iso_ was determined from a measurement in liquid solution [43,45].

The principal values of the on-site *A*^Cu^ HF tensor, the g-tensor and the averaged principal values of the transferred HF tensor for one individual N donor have been determined by the modeling of the spectra. For the modeling, the Hamiltonian in Equation 1 was adopted and the following assumptions have been made [43,45]:

[2]
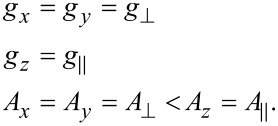


**Figure 4:**
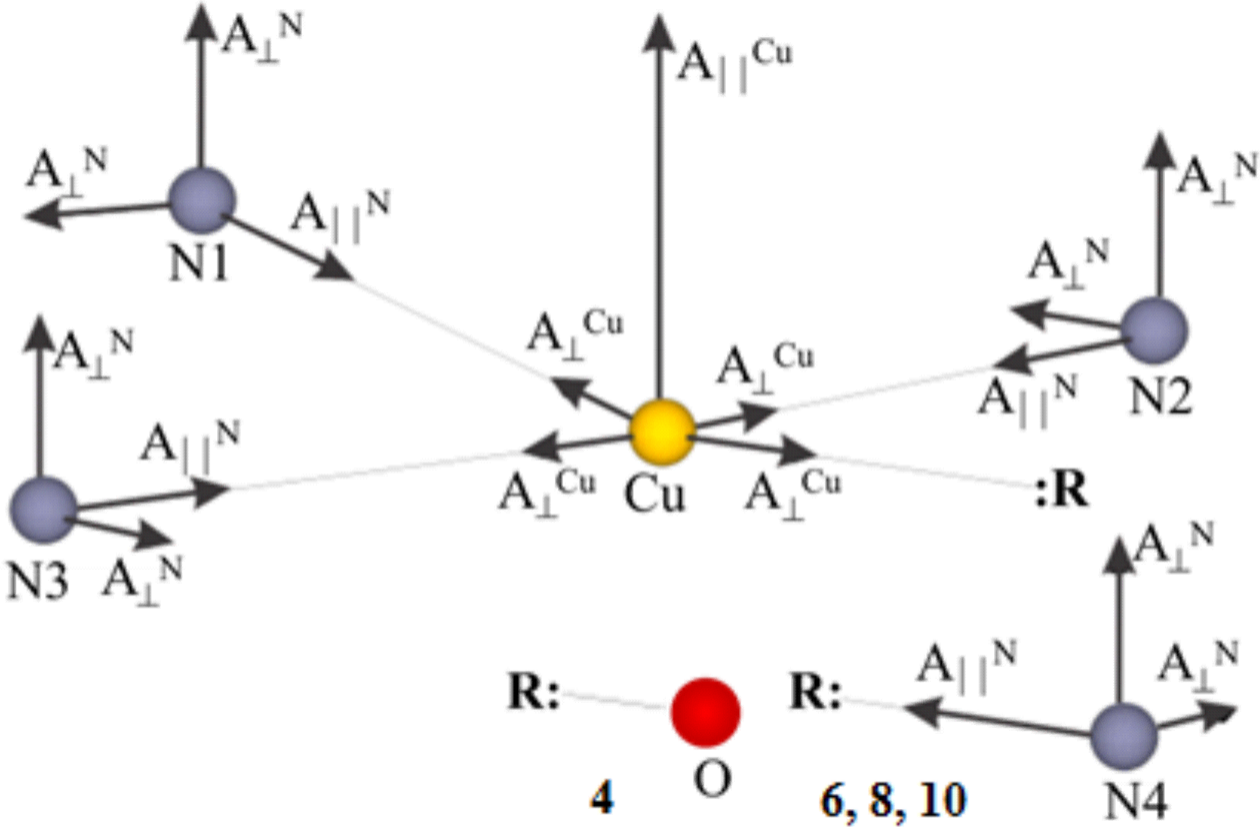
Scheme of the principal axes of the Cu and N HF tensors and the g-tensor. Reproduced with permission from [43], copyright 2015 The Royal Society of Chemistry.

The respective tensor axes of *g*, *A*^Cu^ and *A*^N^ are shown in Figure 4.

**Figure 5:**
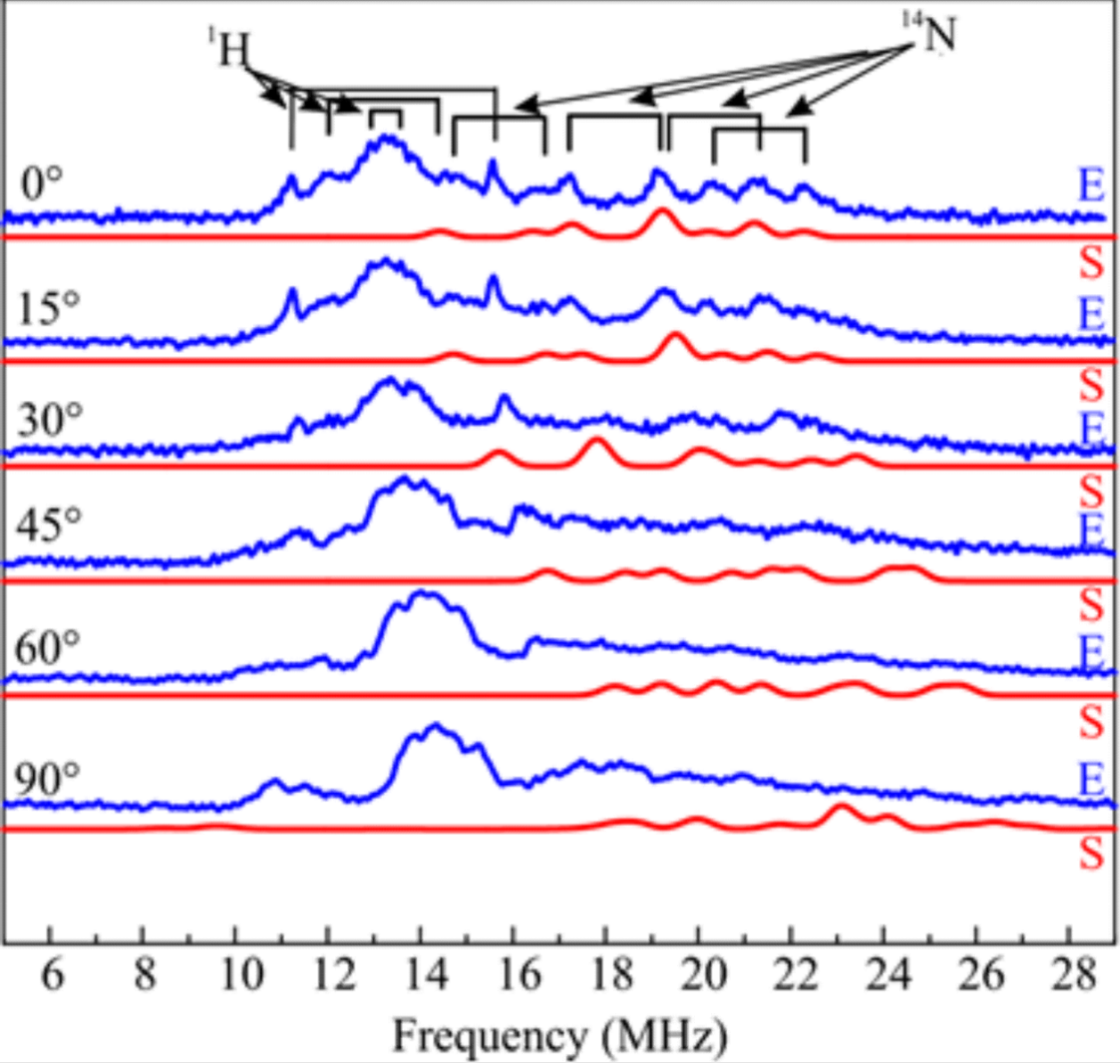
Experimental (E) and simulated (S) Davies ENDOR spectra of **8**@**7** at (ν = 9.56 GHz, *T* = 20 K) at six different orientations of the single crystal in the external magnetic field. The 0° orientation corresponds to the direction of *B*_0_ along the 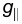-axis of the g-tensor, and the 90° orientation represents the perpendicular direction. Reproduced with permission from [43], copyright 2015 The Royal Society of Chemistry.

Furthermore, pulse Davies electron nuclear double resonance (ENDOR) experiments with an X-band ESR spectrometer Elexsys E580 (Bruker) equipped with the CF935 cryostat and the temperature control unit ITC503 from Oxford Instruments were performed on **8@7** at a temperature of 20 K to obtain additional insights into the HF coupling and to determine the HF tensor of the individual N donor atoms. Conventional ESR experiments do not allow for obtaining such information. The pulse Davies ENDOR technique is based on the detection of the electron spin echo (ESE) [34,39]. The microwave (mw) pulse sequence of this technique consists of three pulses π–τ–π/2–τ–π–ESE that define three periods of the evolution of the spin system (preparation, mixing and detection periods). Changes in the population of the nuclear energy levels induced during the application of an additional radiofrequency pulse in the megahertz range between the first and the second mw pulse reduce the intensity of the ESE signal. Such reductions are measured as a function of the radiofrequency. As an example, experimental and simulated ENDOR spectra of **8@7** at different orientations of the crystal in the magnetic field are shown in Figure 5 [43]. The 0° orientation corresponds to the direction of *B*_0_ along the 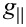-axis of the g-tensor, and the 90° orientation represents the perpendicular direction. The lengths of the first (inversion) π-pulse, and the detection π/2 and π pulses were set to 400 ns, and 16 ns and 32 ns, respectively. The length of the intervening radiofrequency pulse was 7 μs.

The lines in the ENDOR spectrum that arise from the HF coupling of the Cu spin with the nuclear spins of the individual N donor atoms are well resolved. Peaks from protons (^1^H) located at the low frequency part of the spectra can be identified as well. The principal values of the transferred HF tensor of the individual N donor atoms have been obtained from the simulation of the spectra according to the Hamiltonian in Equation 1, taking into account the HF interaction between the ^14^N nuclear spins *I*(^14^N) = 1 of four N-donor atoms and the unpaired electron at the central Cu(II) ion under the assumptions of Equation 2. The values of the g-factor and the on-site HF coupling tensors for Cu(II) were taken from the CW ESR results. The obtained nitrogen HF tensors can be grouped in two groups A and B, and C and D, which were associated with two N_aryl_ and two N_alkyl_ donor atoms, respectively [43]. The values of the tensor components in each pair of groups are quite close. The difference between the groups is considerable. However, the ENDOR measurements indicate some difference in the HF parameters also within each group, which is not evident in the CW ESR results. However, due to a low signal-to-noise (S/N) ratio and the overlap of the lines arising from ^14^N and protons (^1^H) in some orientations, the accuracy of the determination of the N HF tensors with the ENDOR technique was still limited (see below). Possible reasons for the low S/N ratio could be a long duration of the pulse sequence used and temperature instabilities due to sample heating by the radio frequency pulse. In this respect the pulsed electron–electron double resonance (PELDOR) detected NMR (EDNMR) technique turns out to be advantageous as it has a shorter duration of the pulse sequence [38,41]. By performing EDNMR experiments at a higher frequency a possible overlap of a signal by the so**-**called central hole in the EDNMR spectrum can be avoided and a better separation of the lines from ^14^N and ^1^H can be achieved. Therefore, EDNMR experiments have been carried out to verify and refine the HF tensor for the individual N donor atoms of **8@7**, and to accurately determine HF tensor for the individual N donor atoms of **10@9**.

The EDNMR technique uses the excitation of forbidden ESR transitions with the selection rules Δm*_S_* = ±1 and Δm*_I_* = ±1 to measure HF interactions. In this approach, nuclear transition frequencies are measured by exciting two areas within the inhomogeneously broadened ESR line with two mw pulses (preparation and probe pulses). The application of the preparation mw pulse with a frequency ω_mw_^(1)^ corresponding to the low field forbidden transition saturates allowed transitions. These saturations manifest themselves as holes in the ESR line. The pattern of holes is recorded via the integral of the free induction decay (FID) after the selective probe pulse of frequency ω_mw_^(2)^ while varying the frequency difference Δω_mw_ = ω_mw_^(1)^ − ω_mw_^(2)^ giving thus the EDNMR spectrum.

**Figure 6:**
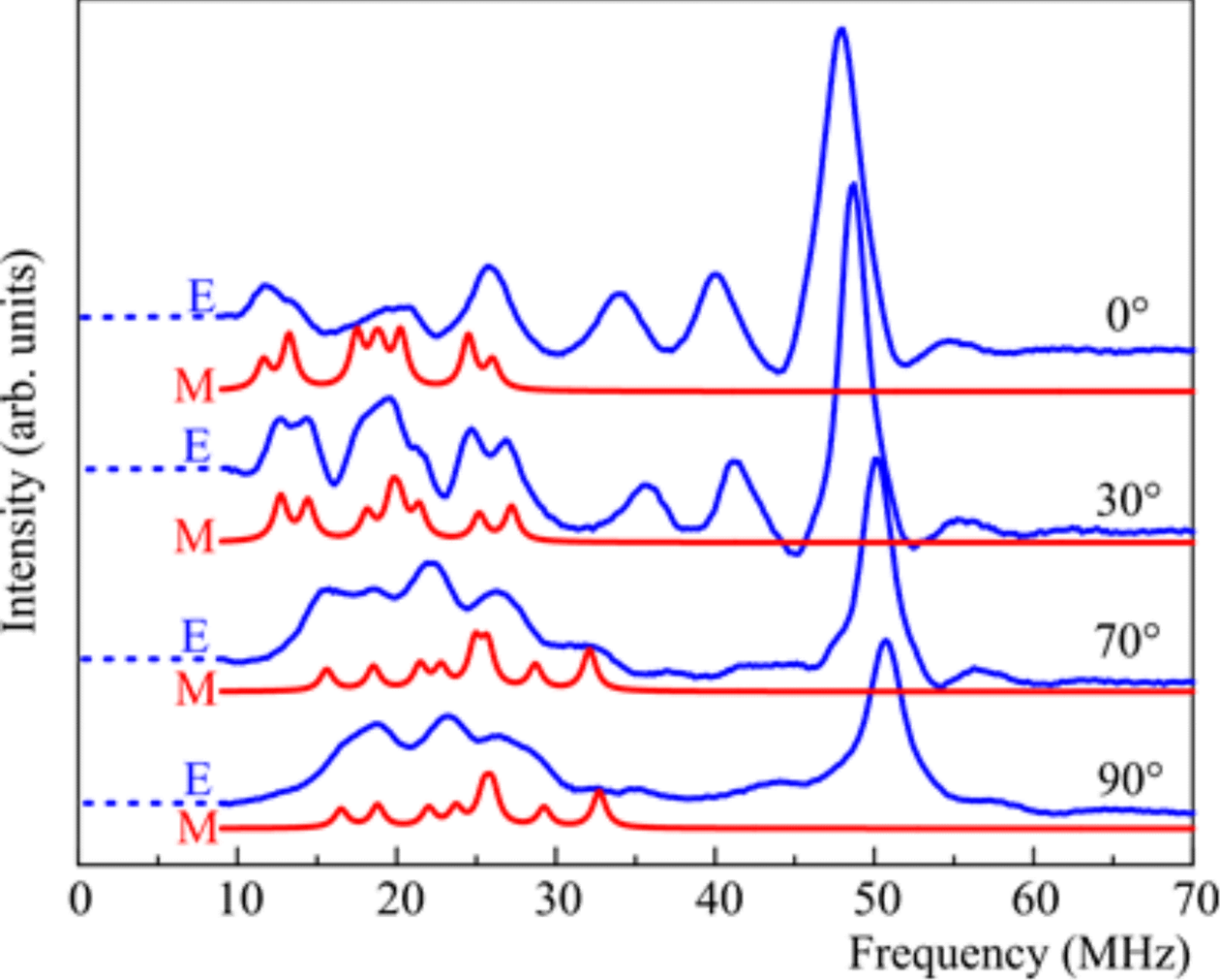
Experimental (E) and modeled (M) EDNMR spectra of **8**@**7** at the Q-band frequency, at *T* = 20 K and at four orientations of the crystal in the external magnetic field *B*_0_. 0^o^ and 90^o^ correspond to *B*_0_ being parallel and perpendicular to the normal of the molecular plane, respectively. Reprinted with permission from [47], copyright 2015 American Chemical Society.

In our experiments reported in [47] the EDNMR spectra of **8@7** and **10@9** at different orientations of the single crystals were recorded with a Q-band ESR spectrometer Elexsys E580 (Bruker) at a temperature of 20 K. The length of the preparation and probe mw pulses were set to 6 μs and 400 ns, respectively. The time delay between these two pulses amounted to 8 μs. Figure 6 shows the experimental and modeled EDNMR spectra of single crystals of **8**@**7** at different orientations of the single crystals. 0° and 90° orientations correspond to *B*_0_ being parallel and perpendicular to the normal of the molecular plane, respectively. ^14^N HF lines are well resolved in all orientations with a substantially better S/N ratio as compared to the pulsed ENDOR spectra of **8**@**7**. This improvement can be explained by the different relaxation paths involved in the two experiments and the smaller number of pulses in the EDNMR protocol. The EDNMR spectra have been modeled with the same approach as used for the analysis of the ENDOR spectra. All experimental lines located in a frequency range of 10–35 MHz are reasonably well reproduced in the simulation and therefore can be assigned to the system of one electron spin *S* = 1/2 of Cu(II) coupled to the ^14^N nuclear spins *I*(^14^N) = 1 of four N donor atoms in the studied complexes. The additional lines located at higher frequencies can arise when other nuclear spins are coupled to the electron spins [47]. At the Q-band frequency, which corresponds to a magnetic field of approximately 1 T, the proton (^1^H) Larmor frequency is ca. 50 MHz. Therefore protons probably also contribute to the line in the EDNMR spectra around 50 MHz.

Like the ENDOR results, the obtained HF tensors indicate two different groups of N ligands classified as groups A and B for **8**. The same observation is obtained for **10** as well. The ^14^N HF constants for group A are close to those of **2** [9]. Therefore, group A could be assigned to N_aryl_ nitrogen donor atoms while group B, with smaller ^14^N HF constants, corresponds to the N_alkyl_ nitrogens of **8** and **10**, respectively.

### Estimates of the electron spin densities

In our works [43,45] as well as in [9], two different models introduced by Maki and McGarvey [48] and Morton and Preston [49] were followed to calculate the spin densities on the Cu(II) ion and N donor atoms of Cu(II) complexes from experimentally obtained HF coupling constants. In the approach of Maki and McGarvey [48], the perpendicular and the parallel Cu HF coupling constants for a complex containing a Cu(II) ion surrounded by four ligands in a square planar configuration are derived and expressed as:

[3]
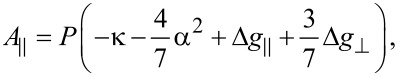


[4]
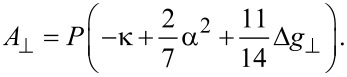


In these expressions, *P*κ is the Fermi contact term with *P*(^63^Cu) = μ_B_*g*_e_μ_n_γ·<r^3^>_3d_ = 1164 MHz, that is, the dipolar HF coupling parameter of the unpaired electron [50], and 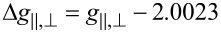. The parameter α^2^ is a covalency parameter that describes the in-plane metal–ligand σ-bonding. The value of α^2^ represent the spin density on the Cu(II) ion (ρ^Cu^ (total)) and was determined by using Equations 1 and 2, and experimentally obtained Cu HF coupling constants for the complexes under study. Furthermore, the procedure of Morton and Preston [49] was used to calculate the spin density on the N donor atoms and the Cu(II) ion. The values obtained for the spin density on the Cu(II) ion were compared with those deduced by the procedure of Maki and McGarvey [48].

According to Morton and Preston [49], the isotropic and anisotropic HF coupling constants for unit spin density on the corresponding s- and p-, d- and f-orbitals have been obtained, respectively. Spin densities on the copper and nitrogen s-orbitals for our complexes are derived as the ratio of the isotropic HF coupling constants obtained experimentally and the theoretical isotropic HF parameters *A*_0_^Cu^ = 5995 MHz and A_0_^N^ = 1811 MHz for unit spin density [49]. The spin density on the p- and d-orbitals of N and Cu, respectively, is proportional to the dipolar HF coupling constant 
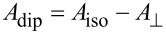
. These contributions are calculated as the ratio of the respective *A*_dip_ derived from the experimental values and the theoretical anisotropic HF parameters 138.8 MHz and 1197 MHz, which are calculated for unit spin density on the nitrogen p- and copper d-orbitals [49], respectively.

### Results of electron spin density measurements

**Figure 7:**
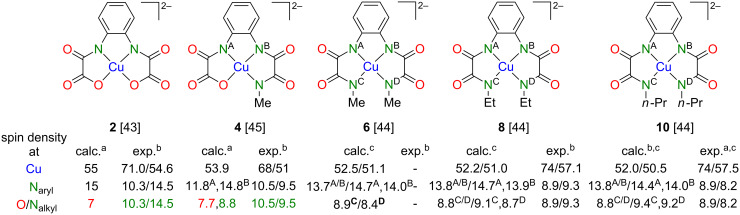
Selected values of calculated versus experimentally determined spin densities of **2**, **4**, **6**, **8**, and **10**. a) Data refer to geometry optimized fragments only. b) The first/second entry refers to data obtained according to the approaches by Maki and McGarvey [48]/Morton and Preston [49]. c) The first/second entry refer to data obtained for geometry optimized complex fragments or to data obtained by using complex geometries from crystallographic characterization, respectively. For further data of **4** cf. [44]. Reproduced with permission from [43], copyright 2015 The Royal Society of Chemistry.

Obtained results of experimentally determined spin density distributions via the approaches by Maki and McGarvey [48] and Morton and Preston [49] according to the above described procedure are summarized in Figure 7. Furthermore and for comparison, DFT-calculated values of **2**, **4**, **6**, **8** and **10** are given as well. One can notice that the experimentally determined spin densities of the Cu(II) ions of **8** and **10** are very similar and exceed the value reported for **2**, while for **4** a lower value compared to **2** was determined. Thus, experimentally obtained data do not follow the suggested tendency that replacing the O donor by N_alkyl_ donor atoms results in a lower spin density on Cu(II) and in higher spin densities at the N_aryl_ donor atoms (Figure 7).

As pointed out in [43], larger HF parameters of tensors A–B (see above) associated with N_aryl_ donor atoms generally indicate a larger spin density *r* on N_aryl_ as compared to that on N_alkyl_ donor atoms, which reveal smaller parameters of the associated HF tensor C. Surprisingly the HF tensor D, which is also related to N_alkyl_, appears to be similar to A–B. This could be a result of an overestimate of the anisotropy of the HF couplings 
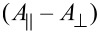
 due to the still somewhat limited accuracy of the performed ENDOR experiments.

**Figure 8:**
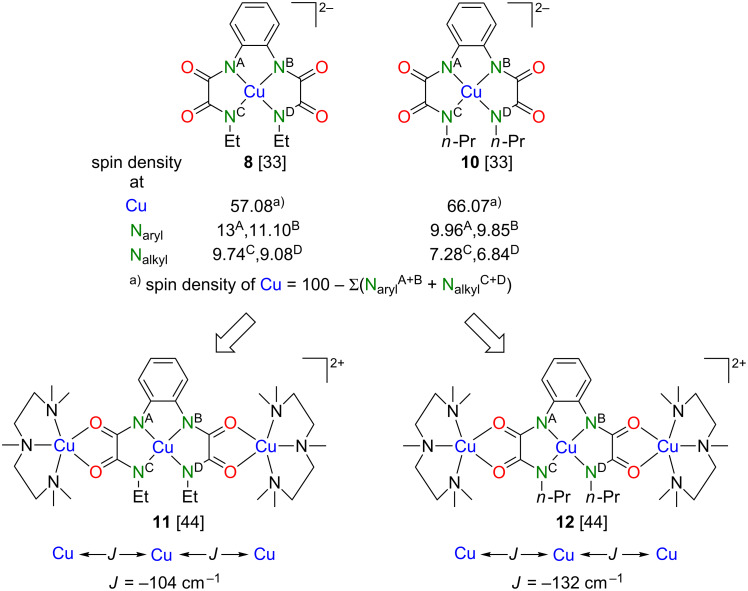
EDNMR-determined spin densities of the N donor atoms of **8** and **10** and *J* values of their corresponding trinuclear complexes **11** and **12**.

In order to demonstrate that the spin densities of abovementioned N donor atoms are indeed different, we carried out additional pulse EDNMR studies of **8** and **10** [47] (Figure 6). The result of this study revealed unambiguously that the spin densities of the N_aryl_ donor atoms of **8** and **10** are significantly larger compared to those of the N_alkyl_ donor atoms (Figure 8). Moreover, it could be demonstrated that all four N donor atoms of both **8** and **10** possess different spin densities, as revealed by quantum chemical calculations (Figure 7, Figure 8 and [43]). The EDNMR experiments did, however, not allow for the determination of the spin densities at the Cu(II) ions of **8** and **10**. In order to give a rough estimate for a comparative discussion, they have been calculated as described in Figure 8. According to the much lower spin density of Cu(II) in mononuclear **8** (ca. 57%) compared to **10** (ca. 66%) we expected a significantly higher *J* value for trinuclear **11** compared to **12**. However, the opposite situation has been determined experimentally (Figure 8 and [43]). This counterintuitive result has been explained by considering both differences of the local geometries of the terminal and central Cu(II) ions of **8** and **10**.

We already noticed with surprise the differences of the coordination behavior of the terminal [Cu(pmdta)]^2+^ complex fragments to binuclear bis(oxamidato)-type complexes as represented by **8** and **10** compared to type-**II** complexes displayed in Scheme 1 [43]. The different coordination behavior is expected to have an impact on the magnetic superexchange coupling pathways in corresponding trinuclear complexes. As a consequence, we realized that there are further parameters to be considered when deriving a conclusion whether the spin densities of mononuclear type-**II** or bis(oxamidato) complexes could be a measure of *J* values of their corresponding trinuclear complexes. In this context we note, that determination of the spin densities from the HF parameters should be considered as a reasonable method to obtain qualitative estimates and tendencies to be compared and discussed together with the DFT results and to be critically evaluated with respect to geometrical features of the studied molecular complexes. In particular, the approach of Maki and McGarvey [48] implies a square planar geometry of the molecule, a condition that is not always fulfilled. A limitation of the model by Morton and Preston [49] is that it presumes calculation of the HF constants for a free atom. With this in mind, it is indeed reasonable to conclude that the transfer of the spin density from the metal ions to the bonding ligands is certainly an important but not yet the decisive factor that determines the strength of the superexchange interactions in the trinuclear complexes **11** and **12**. In this particular case, it is most likely the steric factors that eventually yield the stronger antiferromagnetic exchange interaction in **12** as compared to **11**.

This presentation of our results should not end without reference to further studies of such diamagnetically diluted single crystals. We have recently shown the means of manipulating and enhancing the electron spin coherence of **2@1** and **10@9** at different temperatures by applying special microwave pulse sequences in an ESR experiment [51]. We aim to obtain with such experiments further insights into an understanding of mononuclear type-**II** and bis(oxamidato) complexes with respect to their electron spin dynamics, spin coherence and relaxation processes as well as their possible applications in molecular electronic devices.

## Conclusion

Is there an interplay between the spin density distribution of mononuclear bis(oxamato) type-**II** and related complexes and the magnetic superexchange interactions of their related trinuclear type-**III** complexes? Our results support this hypothesis, although they clearly indicate that many further experiments are required to establish this interplay. For example, the O donor atoms of type-**II** complexes should be all substituted by N_alkyl_, or alternatively by S donor atoms. Such substitutions are expected [32] to modify the spin density distribution of the mononuclear building blocks to a much higher extent compared to our here reported initial studies. As the transfer of the spin density along the bridging groups is a prerequisite for supermagnetic exchange couplings, such a modification of the mononuclear complexes should result in higher *J* couplings accordingly. Here, the specifics of the bonding geometry and its implications for the superexchange interactions have to be considered seriously.

On the other hand, we reported to which extent the materials made available, especially the diamagnetically diluted single crystals, prompted us to apply complex pulse ESR techniques for a better understanding of the physical properties of our compounds. This progress leads to the request for advanced types of materials. It would be thus challenging to replace the paramagnetic terminal [Cu(pmdta)]^2+^ complex fragments of **11** and **12** by diamagnetic [Zn(pmdta)]^2+^. Such heterotrinuclear Zn(II)Cu(II)Zn(II) complexes would allow us to determine the spin density distribution of the central building blocks “in action”, that is, when contributing to magnetic exchange couplings especially in the case that the Zn(II)Cu(II)Zn(II)-type complexes are isomorphic to **11** and **12**. On first of such efforts we already report in this Thematic Series, namely the synthesis and characterization of heterotrinuclear Cu(II)Ni(II)Cu(II) bis(oxamidato)-type complexes [52].
